# Research on the Preparation of Polylactic Acid/Bamboo Fiber Composite Materials and Their 3D Printing Process

**DOI:** 10.3390/ma19050851

**Published:** 2026-02-25

**Authors:** Zhenxiao Xu, Zixin Hu, Bin Wang, Sisi Wang

**Affiliations:** College of Engineering, Zhejiang Normal University, Jinhua 321004, China; 17857586269@163.com (Z.X.); 13956945533@163.com (Z.H.)

**Keywords:** bamboo fiber, interfacial modification, 3D printing, impact properties, orthopedic braces

## Abstract

**Highlights:**

**What are the main findings?**

- Bamboo fiber-reinforced PLA/PBAT composites achieve high impact strength.
- Silane treatment enhances fiber–matrix dispersion, boosting composite toughness.
- Foot braces fabricated at 35 mm/s demonstrate high impact strength and structural integrity.

**What are the implications of the main findings?**

- Provides a scalable interfacial modification strategy for natural fiber composites.
- Expands bio-based material applications in orthopedic braces.
- Establishes a process for high-performance 3D-printed braces, advancing personalized medicine.

**Abstract:**

The increasing need for lightweight, personalized, and sustainable orthopedic braces has motivated the development of bamboo fiber (BF)-reinforced polylactic acid (PLA) composites. In this study, BF/PLA composites were prepared by melt blending. The effects of polybutylene adipate terephthalate (PBAT) toughener, BF content, and a silane coupling agent on the mechanical properties were evaluated, along with their suitability for 3D printing foot braces. The results showed that at a PLA/PBAT mass ratio of 85/15 and a bamboo fiber content of 10 wt.%, the impact strength of the composite reached 7.7 kJ/m^2^. Silane treatment of BF further improved the impact strength, with a maximum value of 11.3 kJ/m^2^ achieved at a silane/BF mass ratio of 2/98. The optimized composite exhibited good printability across nozzle temperatures of 190–210 °C. Printing speed significantly influenced the process; a speed of 35 mm/s enabled successful fabrication of the foot brace, whereas higher or lower speeds led to model collapse due to overheating or cracking caused by insufficient interlayer adhesion. This study successfully developed a bamboo fiber-reinforced PLA composite suitable for 3D printing of orthopedic braces and identified the optimal 3D printing process parameters.

## 1. Introduction

Since the beginning of the 21st century, the world has faced increasingly severe challenges from plastic pollution and energy crisis. It is estimated that approximately 8 million tons of plastic waste enter the oceans each year, where, due to a degradation cycle lasting centuries, they cause long-term damage to ecosystems [1]. Concurrently, the production of petroleum-based materials continues to consume non-renewable resources and contribute to carbon emissions [2]. Against this backdrop, bio-based biodegradable materials have attracted significant attention for their low-carbon and renewable characteristics.

Polylactic acid (PLA), as a representative bio-based polymer, is widely regarded as an ideal alternative to conventional plastics owing to its renewable feedstock and favorable processability [3]. However, its mechanical properties—such as impact strength and heat deflection temperature—remain relatively poor, which limits its practical applications [4]. Introducing natural fiber reinforcements can improve material performance while maintaining biodegradability. Bamboo fiber (BF), a renewable resource with high specific strength and low density, not only enhances the mechanical properties of PLA composites but also reduces their life-cycle carbon emissions [5]. Studies indicate that bamboo fiber-reinforced composites exhibit approximately 20% higher specific stiffness and 40% lower carbon emissions compared to glass-fiber counterparts [6]. Nevertheless, issues such as high moisture absorption, poor thermal stability, and inadequate interfacial compatibility still restrict the wider application of bamboo fibers [7].

Various modification methods have been developed to address these limitations. For instance, Wang et al. [8] obtained high-strength bamboo fiber bundles through saturated steam pretreatment at 180 °C combined with mechanical rolling. Chiu et al. [9,10,11,12] improved interfacial properties and bonding strength via alkaline treatment and chemical modification. Song et al. [13] used silane KH550 to treat bamboo flour, leading to enhanced crystallinity and thermal performance of the resulting composites. Despite these advances, bamboo fiber/PLA composites still face several challenges: interfacial optimization strategies remain relatively singular, making multi-scale synergistic reinforcement difficult to achieve; the influence of printing parameters on performance remains poorly quantified; and studies on long-term durability are still insufficient [14,15,16].

Compared with conventional molding techniques, fused deposition modeling (FDM) offers a novel pathway for the customized fabrication of complex structural components. This technology enables direct generation of intricate geometries from 3D CAD models, demonstrating unique advantages in fields such as lightweight aerospace parts and biomedical scaffolds [17]. It thus provides a transformative means for the customized design and complex-shape fabrication of composite materials [18]. Unlike traditional injection molding, 3D printing requires no molds, reduces material waste, supports topology-optimized structures, and significantly improves specific strength [19].

Recent advances have highlighted the potential of 3D-printed PLA composites in medical applications, particularly in orthopaedic implants, tissue engineering, and customised medical devices [20]. Nevertheless, bamboo fiber-reinforced PLA for 3D-printed orthopaedic devices remains largely unstudied, and critical issues such as inadequate interfacial bonding, limited impact toughness, and undefined printing parameters have yet to be systematically addressed.

This study aims to develop high-toughness BF/PLA composites suitable for 3D printing. By modifying the fibers with a silane coupling agent to further enhance toughness and optimizing the printing process parameters, the research ultimately targets the application of these materials in the field of orthopedic devices.

## 2. Materials and Methods

### 2.1. Materials

The PLA (REVODE190) pellets were supplied by Zhejiang Hisun Biomaterials Co., Ltd. (Zhejiang, China). It has a theoretical density of 1.25 ± 0.05 g/cm^3^ and a melting temperature of approximately 170–180 °C. The polybutylene adipate terephthalate (PBAT) pellets were produced by Kingfa Sci & Tech Co., Ltd. (Guangzhou, China), with the grade NC901. Bamboo Fiber (80 mesh) was produced by Jiangxi Jintai Bamboo Fiber Technology Co., Ltd. (Pingxiang, China), Epoxidized Soybean Oil (KD-82) was produced by Shenzhen Kaiqi Chemical Co., Ltd. (Shenzhen, China), and the Silane Coupling Agent (KH570) was produced by Shanghai Macklin Biochemical Technology Co., Ltd. (Shanghai, China).

### 2.2. Surface Modification of Bamboo Fibers

Bamboo fibers were surface-modified with KH570. The fibers were first mixed with deionized water in a container and heated at 80 °C under stirring for 3h to remove lignin and surface impurities. After washing and drying, the bamboo fibers were separately immersed in KH570 solutions with concentrations of 1 wt.%, 2 wt.%, and 3 wt.%. Each mixture was maintained at 80 °C with continuous stirring for 4h. The treated fibers were then filtered, dried, and further oven-dried for 24h to obtain the modified bamboo fibers.

### 2.3. Sample Preparation

#### 2.3.1. Preparation of Twin-Screw Extruded Pellets

PLA/PBAT blends and bamboo fibers (BF) were compounded and pelletized using a twin-screw extruder (SHJ-35A, Nanjing Shengchi Rubber Machinery Manufacturing Co., Ltd., Nanjing, China). Composites with varying PLA/PBAT ratios were prepared to identify the optimal blend composition. To ensure stable extrusion and prevent premature melting and adhesion at the feed inlet, the temperature was set to 130 °C at the feed zone and gradually increased to 200 °C toward the outlet. The specific temperature profile along the extruder barrels was set at 130, 170, 190, 200, 200, 200, 200, 200, 200, and 200 °C. The resulting composite pellets were dried in an oven at 80 °C for 24h to ensure complete moisture removal, yielding the final BF/PLA composite material.

The experimental procedure was conducted in two stages. First, unmodified bamboo fiber composites with different PLA/PBAT weight ratios were prepared to determine the optimal polymer matrix formulation. Subsequently, based on this optimal ratio, composites incorporating bamboo fibers modified with different concentrations of KH570 silane coupling agent were fabricated. The specific formulations of all prepared composites are detailed in Table 1.

#### 2.3.2. Fabrication of Test Specimens

Standard mechanical test specimens were fabricated using a screw extrusion-based 3D printer (4-Axis Printer, Zhejiang Chaoling Intelligent Technology Co., Ltd., Jinhua, China). The specimen geometry adhered to the following standards: Type I tensile bars (ASTM D638-10 [21]), flexural bars (ASTM D7264 [22]), and notched impact bars (ASTM D256 [23]). The printing parameters were set as follows: nozzle diameter of 2 mm, printing temperature of 200 °C, heated bed temperature of 60 °C, printing speed of 60 mm/s, layer height of 0.4 mm, and 100 wt.% infill density. The printing direction was aligned parallel to the *x*-axis of the printer, which corresponds to the axis of tensile testing.

### 2.4. Testing and Characterization

Contact Angle Measurements (FCA2000A1, Shanghai Aifes Precision Instruments Co., Ltd., Shanghai, China). Water contact angles were measured to evaluate the surface wettability of the materials using a contact angle analyzer. Deionized water (1.5 µL droplets) was deposited onto the sample surface using a microsyringe, and the static sessile drop method was employed. At least five measurements were taken at different locations on each sample to ensure reproducibility.

Mechanical Property Testing (Shenzhen Suns Technology Stock Co., Ltd., Shenzhen, China). Tensile and flexural tests were performed on a universal testing machine (UTM4204) at a crosshead speed of 2 mm/min. Notched Izod impact strength was determined using an impact tester (PTM7151) with a pendulum energy of 5.5 J. To minimize experimental variability, a minimum of five specimens were tested for each condition, and the results are reported as the mean value with standard deviation.

Microscopic Morphology Analysis (Sigma 300, Carl Zeiss AG, Oberkochen, Germany). The fracture surfaces of the specimens were examined using a scanning electron microscope (SEM) to analyze the microstructure and interfacial adhesion. Prior to imaging, the samples were sputter-coated with a thin layer of gold to enhance conductivity. Observations were carried out under vacuum at an accelerating voltage of 5 kV.

Thermal Behavior Analysis (DSC 214, NETZSCH Scientific Instruments Trading (Shanghai) Ltd., Shanghai, China). Differential scanning calorimetry (DSC) was employed to evaluate the melting and crystallization behavior of the composites. Samples weighing 5–10 mg were sealed in aluminum pans and tested under a nitrogen atmosphere to prevent thermal degradation. The thermal protocol was as follows: first, the samples were heated from 30 °C to 210 °C at a rate of 10 °C/min and held at 210 °C for 5 min to erase any prior thermal history. Subsequently, the samples were cooled to 30 °C at 10 °C/min. Finally, a second heating scan was performed at 10 °C/min to 210 °C. The degree of crystallinity (*X_c_*) was determined from the second heating DSC thermogram according to the following equation:

(1) $$X_{c}\;=\;\frac{\Delta H_{m}\;-\;\Delta H_{cc}}{\Delta H_{m0}\;\times \;\omega }\;\times \;100\%$$

where Δ*H_m_* is the enthalpy of melting (J/g), Δ*H_cc_* is the enthalpy of cold crystallization (J/g), Δ*H_m_*_0_ is the theoretical enthalpy of fusion for 100% crystalline material (J/g), and *ω* is the mass fraction of the respective component. The Δ*H_m_*_0_ values used for PLA and PBAT were 93.6 J/g and 114 J/g, respectively.

## 3. Results and Discussion

### 3.1. Water Contact Angle Analysis

The surface of bamboo fibers (BF) was modified with KH570 to reduce their inherent hydrophilicity. The strong hydrophilic nature of BF primarily stems from the abundance of hydroxyl groups on its surface. The trimethoxysilane groups in KH570 can undergo a hydrolysis-condensation reaction with these surface hydroxyls. This process effectively reduces the number of hydrophilic groups and concurrently forms a hydrophobic organosilane layer on the fiber surface, thereby enhancing the interfacial properties of the composite material [24].

**Figure 1:**
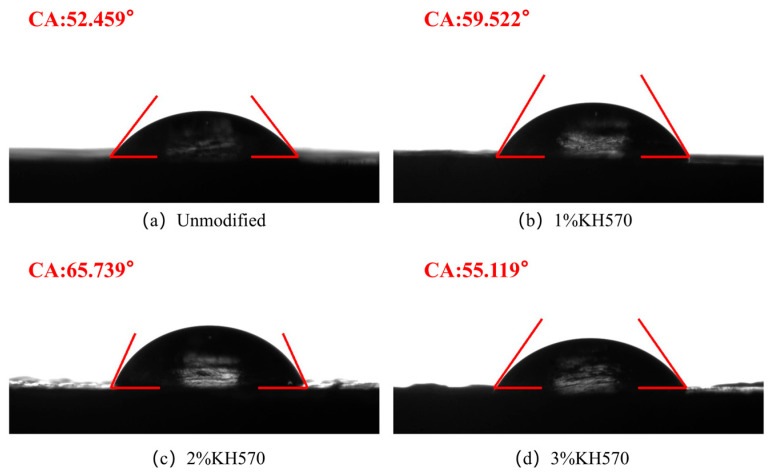
Water contact angles of BF/PLA composites.

The efficacy of the modification was evaluated through water contact angle measurements, with the results presented in Figure 1. Figure 1a–d display the contact angles for the unmodified BF/PLA composite and those modified with 1 wt.%, 2 wt.%, and 3 wt.% KH570, respectively. The contact angle for the unmodified composite was 52.459°. After treatment with 1 wt.%, 2 wt.%, and 3 wt.% KH570, the contact angles increased to 59.522°, 65.739°, and 55.119°, respectively. This results indicates that KH570 treatment effectively improved the hydrophobicity of the composites. The most significant improvement was observed with the 2 wt.% KH570 modification, which yielded the highest contact angle, suggesting an optimal degree of surface modification and a marked improvement in the composite’s moisture resistance at this concentration.

When the KH570 concentration was increased to 3 wt.%, the contact angle decreased. This decline may be attributed to the self-condensation of excess silane molecules after hydrolysis, which reduces their effective bonding with the hydroxyl groups on the bamboo fiber surface, thereby diminishing the interfacial modification effect. Therefore, controlling the silane coupling agent concentration is essential for achieving the desired hydrophobic performance.

### 3.2. Analysis of Mechanical Properties of the Composites

Mechanical properties serve as critical metrics for evaluating the application potential of composite materials. In this study, standard test specimens were fabricated via 3D printing. Compared to components produced by conventional thermoplastic processing, 3D-printed parts often contain inherent structural defects, which impose certain limitations on their mechanical performance. Targeting the specific requirements of orthopedic braces, this work focuses on developing bamboo fiber-reinforced composites with enhanced impact strength. However, in most composite systems, improvements in toughness are frequently accompanied by a reduction in strength and stiffness [25]. Consequently, this study aims to enhance the material’s toughness while striving to preserve its inherent strength and rigidity, thereby achieving a synergistic optimization of its overall mechanical performance.

#### 3.2.1. Mechanical Properties of Unmodified BF/PLA Composites

**Figure 2:**
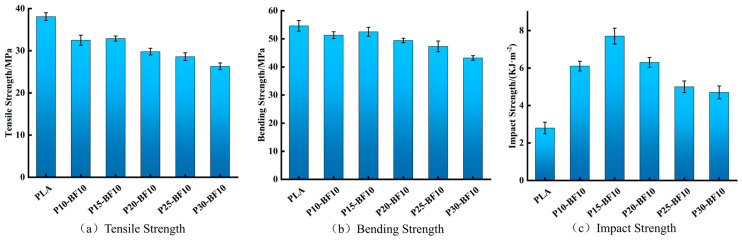
The mechanical properties of unmodified composites.

Five BF/PLA composites with a fixed bamboo fiber content of 10 wt.% and varying PLA/PBAT ratios were prepared. Standard mechanical specimens were fabricated using a screw-extrusion 3D printer, and their mechanical properties are presented in Figure 2.

As shown in Figure 2, the tensile strength of the composites generally decreased with increasing PBAT content, yet remained around 30 MPa. Compared with pure PLA, the tensile strength decreased by approximately 16%, which is attributed to the inherent trade-off where PBAT enhances toughness while reducing strength and stiffness. The flexural strength also gradually declined with higher PBAT content. However, the flexural strength of pure PLA, PLA/PBAT (90/10), and PLA/PBAT (85/15) systems remained around 50 MPa, indicating no significant reduction until the PBAT content was further increased.

Regarding impact performance, the notched impact strength first increased and then decreased with increasing PBAT content. All formulations exhibited higher impact strength than pure PLA. The maximum impact strength of 7.7 kJ/m^2^ was achieved at a PLA/PBAT ratio of 85/15, representing a 275% increase compared to pure PLA (2.8 kJ/m^2^). This optimal ratio corresponds to the most uniform fiber dispersion observed in SEM images of fracture surfaces. Although PBAT effectively improved toughness, excessive amounts led to extrusion instability during 3D printing due to increased elasticity, resulting in printing defects and a consequent decline in impact strength.

These results indicate that the addition of PBAT led to a slight reduction in tensile and flexural strength, but this decrease remained within acceptable limits. In contrast, the impact strength of the material was significantly enhanced. Based on a comprehensive evaluation of the mechanical properties, the PLA/PBAT ratio of 85/15 achieved an optimal balance among strength, stiffness, and toughness, establishing it as the baseline formulation for subsequent modification experiments.

#### 3.2.2. Mechanical Properties of Modified BF/PLA Composites

Based on the optimized PLA/PBAT blend ratio, this study further employed the silane coupling agent KH570 at different concentrations to modify the bamboo fiber surface, aiming to enhance interfacial compatibility with the polymer matrix. To systematically evaluate the effect of fiber content, experimental groups with bamboo fiber loadings of 5 wt.%, 10 wt.%, and 15 wt.% were established for comparative mechanical analysis.

**Figure 3:**
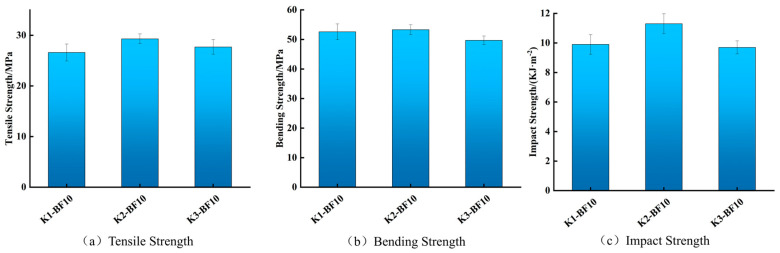
Mechanical properties under different concentrations of KH570.

Figure 3 shows that the 2 wt.% KH570 treatment yielded slightly improved tensile and flexural properties relative to other silane concentrations, while delivering a marked increase in impact strength (11.3 kJ/m^2^). This value substantially exceeds the 7.8 kJ/m^2^ reported for an unmodified BF/PLA composite in the literature [26]. This represents a 12 wt.% and 13 wt.% increase over the groups treated with 1 wt.% and 3 wt.% KH570, respectively, and about 404% improvement compared to pure PLA. These results confirm the successful surface modification of bamboo fibers by KH570. The mechanism involves the hydrolysis of silane to form silanols, which react with hydroxyl groups on the bamboo fiber surface, establishing covalent bridges. This enhances the interfacial bonding between the fibers and the PLA matrix, thereby improving stress transfer efficiency and achieving a toughening effect. The superior performance of the 2 wt.% KH570 modification over the 3 wt.% group is attributed to an excess of unreacted silane residues at the higher concentration. These residues can form an uneven silane layer on the fiber surface, which may hinder effective fiber–matrix bonding and induce localized fiber embrittlement, negatively impacting the composite’s toughness [27].

**Figure 4:**
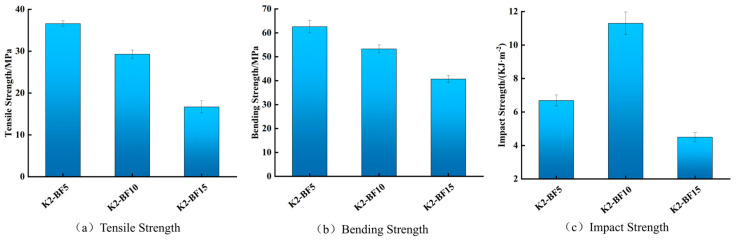
The mechanical properties under different fiber contents.

Given the optimal overall performance observed in the 2 wt.% KH570 treatment group, this study further fabricated composites with bamboo fiber contents of 5 wt.%, 10 wt.%, and 15 wt.%. Their mechanical properties are presented in Figure 4. Regarding tensile and flexural properties, the group with 5 wt.% bamboo fiber content exhibited the highest strengths, reaching 37.4 MPa and 62.5 MPa, respectively, which were slightly higher than those of pure PLA. In contrast, the 15 wt.% content group showed the lowest performance, significantly below that of pure PLA. Meanwhile, all other formulations demonstrated flexural and tensile strengths exceeding those recently reported for a modified BF/PLA composite (49.01 MPa and 23.42 MPa, respectively) [28]. This trend reflects the tendency of bamboo fibers to agglomerate at higher contents, leading to poorer dispersion and reduced matrix wettability, which increases porosity and diminishes mechanical performance. In terms of impact strength, the 10 wt.% bamboo fiber group performed best, while the 15 wt.% group performed worst. This indicates that at 5 wt.% fiber content, the interfacial stress transfer efficiency is insufficient, whereas at 15 wt.% content, fiber agglomeration induces stress concentration. At 10 wt.% content, uniform fiber dispersion enables the formation of a dense reinforcement network that effectively bears and transfers critical stresses, thereby significantly enhancing the composite’s toughness [29].

Based on the mechanical performance results summarized above, it can be concluded that the BF/PLA composite modified with 2 wt.% KH570 and containing 10 wt.% bamboo fiber exhibits optimal impact strength along with favorable tensile and flexural strength. This provides a critical experimental foundation for its subsequent application in 3D printing of orthopedic braces.

### 3.3. Fracture Surface Morphology Analysis of the Composites

Scanning electron microscopy (SEM) was used to examine the fracture surfaces of the specimens, providing direct insight into the interfacial bonding between the matrix and the reinforcement. A well-bonded interface facilitates stress dissipation and promotes effective load transfer from the matrix to the fibers. According to interfacial toughening theory, strong interfacial bonding facilitates efficient stress transfer and activates multiple energy dissipation mechanisms, such as crack deflection, fiber pull-out, and matrix plastic deformation, thereby significantly improving impact toughness [30]. However, pores located within fiber bundles or near the interface substantially reduce the effective load-bearing area of the matrix and serve as preferred sites for crack initiation, accelerating microcrack nucleation and propagation even under low stress. Furthermore, the mechanical performance of short-fiber-reinforced composites is strongly governed by the fiber length distribution and its relation to the critical fiber length. Fracture surface morphology thus provides critical evidence of interfacial bonding quality and failure mechanisms [31].

**Figure 5:**
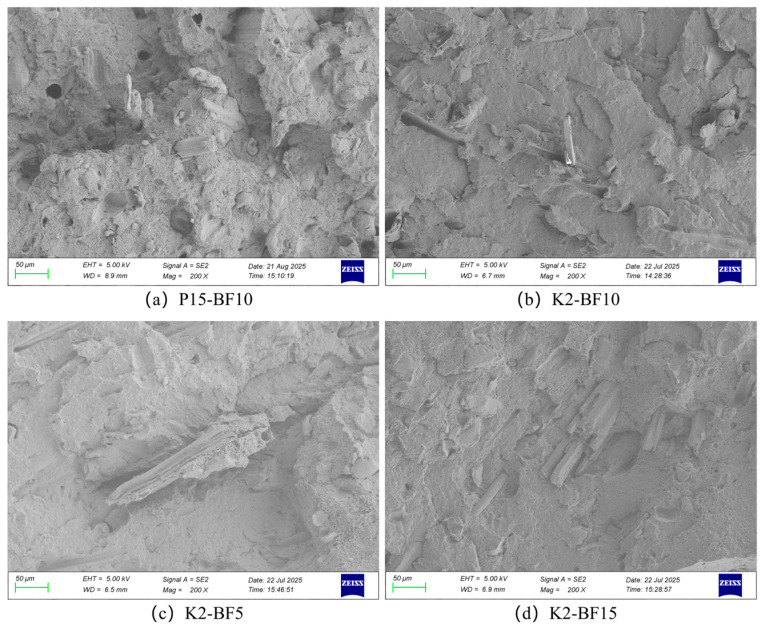
SEM images of sample section.

Figure 5a shows the fracture surface of the PLA/PBAT (85/15) composite containing 10 wt.% unmodified bamboo fiber. Localized fiber agglomeration and numerous voids are evident, indicating that although fiber dispersion is improved at this specific ratio compared to other unmodified formulations, the interfacial adhesion between the fibers and the matrix remains relatively weak and requires further optimization.

Figure 5b–d present the fracture morphologies of composites modified with 2 wt.% KH570 at different bamboo fiber contents (5 wt.%, 10 wt.%, and 15 wt.%, respectively). Silane treatment significantly enhances fiber–matrix adhesion, as reflected by the substantial reduction in void size and quantity, along with a more uniform fiber length distribution. These microstructural improvements are consistent with the enhanced impact strength discussed in Section 3.2.2. Among the modified groups, the composite containing 10 wt.% bamboo fiber exhibits the most homogeneous fiber dispersion, the finest and sparsest voids, and pronounced wave-like plastic deformation features. In contrast, at 15 wt.% fiber content, although interfacial voids are further minimized, severe fiber agglomeration reappears, leading to localized stress concentration and a consequent deterioration in mechanical performance.

In summary, when the bamboo fiber content is 10 wt.% and the fibers are treated with 2 wt.% KH570, they are uniformly dispersed within the PLA/PBAT matrix without noticeable phase separation, and the interfacial bonding between the two phases is excellent. Under these conditions, the bamboo fibers effectively fulfill their reinforcing and toughening roles, resulting in the optimal overall mechanical performance of the composite.

### 3.4. 3D-Printed Orthopedic Braces

**Figure 6:**
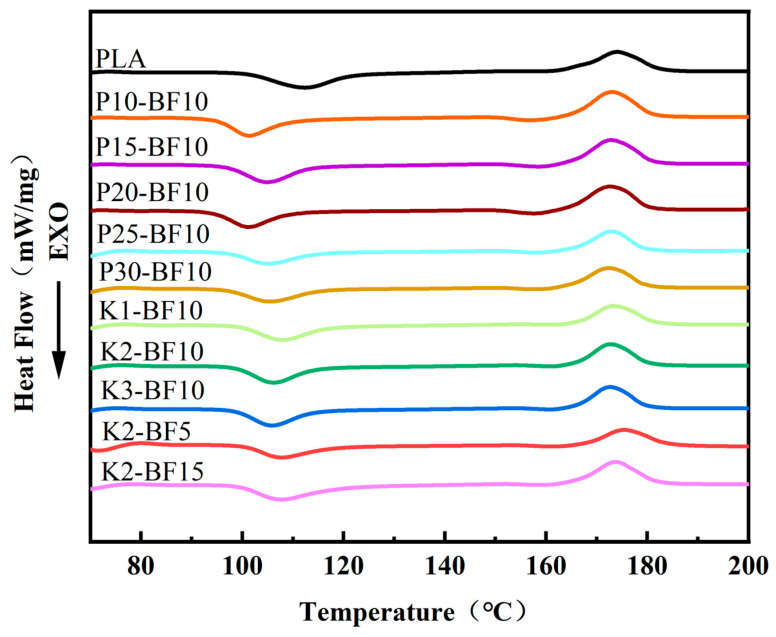
DSC thermogram.

DSC measurements were conducted to facilitate the selection and analysis of 3D printing parameters; the corresponding data are summarized in Figure 6 and Table 2.

Table 2 shows that the melting temperatures of all samples exhibit negligible variation. In contrast, the degree of crystallinity declines steadily upon KH570 incorporation, attaining its lowest value of 5.92% at a silane concentration of 2 wt.%. Such a reduction in crystallinity is conducive to improved moldability and dimensional fidelity during 3D printing.

**Figure 7:**
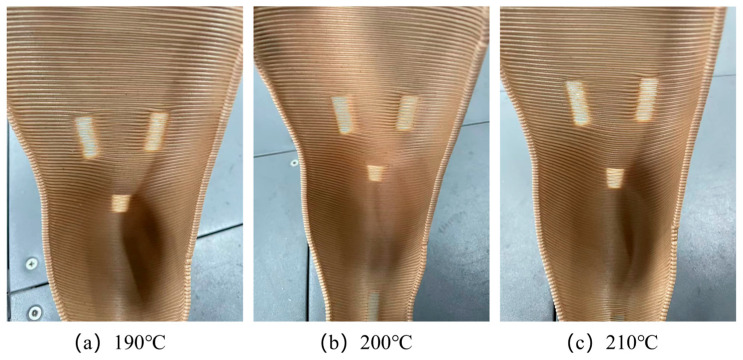
Orthopedic braces images at different printing temperatures by using 10 wt.% BF/PLA.

**Figure 8:**
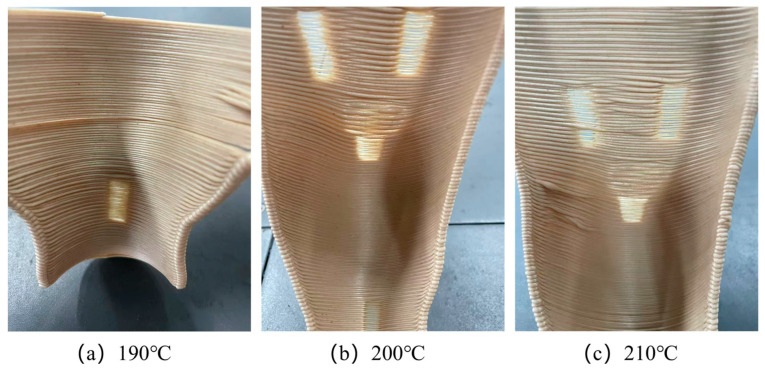
Orthopedic braces images at different printing temperatures by using 5 wt.% BF/PLA.

Building on the enhanced toughness achieved through modified bamboo fibers, this study further investigated the optimization of 3D-printing parameters for orthopedic braces applications. Figure 7 and Figure 8 present photographs of foot braces printed from BF/PLA composites containing 5 wt.% and 10 wt.% bamboo fibers (modified with 2 wt.% KH570) at different nozzle temperatures (190 °C, 200 °C, and 210 °C). The results show that the composite with 10 wt.% bamboo fiber content not only exhibited the best toughness but also printed successfully at all three temperatures, yielding intact products with no obvious defects. In contrast, the composite with 5 wt.% bamboo fiber, despite its higher tensile and flexural strength, showed insufficient toughness and was prone to structural defects during printing, compromising the quality of the final part. At 5 wt.% fiber content, the reinforcement phase remains below the percolation threshold and is insufficient to establish a continuous stress-transfer network. Although the composite exhibits marginally higher tensile and flexural strengths than 10 wt.% fiber content, the absence of an interconnected fiber network restricts key energy dissipation mechanisms. This failure behavior is consistent with observations in short-fiber-reinforced polymer systems, where low fiber content leads to failure dominated by fiber pull-out and matrix folding, limiting energy absorption [32]. Consequently, the material remains brittle and susceptible to interlayer cracking during 3D printing. In the present system, the 10 wt.% fiber content reaches a critical threshold at which fibers are uniformly dispersed and begin to form an interconnected network. Increasing fiber volume fraction has been shown to directly enhance damage initiation strengths and energy dissipation capacity, supporting the observed transition from brittle to toughened behavior at this optimal loading level [33].

**Figure 9:**
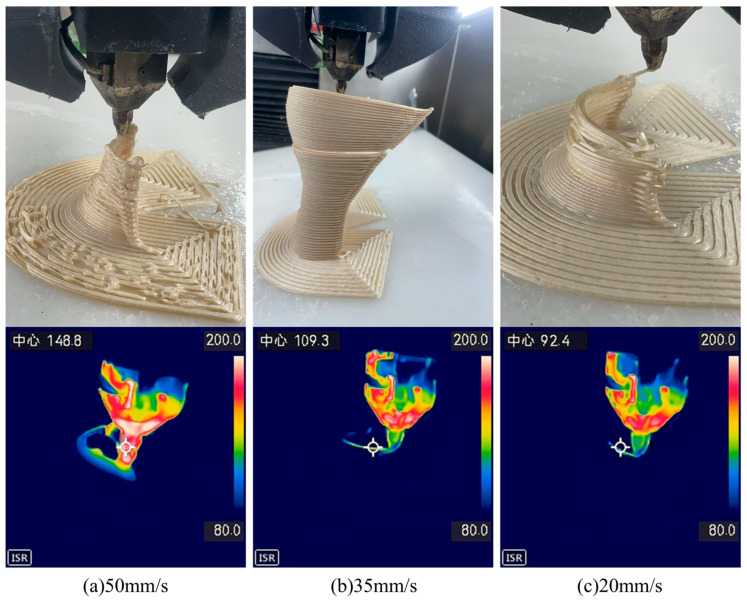
Orthopedic braces images at different printing speeds by using 5 wt.% BF/PLA. Here, Text in the upper left corner refers to the geometric center of the circle indicated by the symbol, and the following number denotes the temperature at that location.

**Figure 10:**
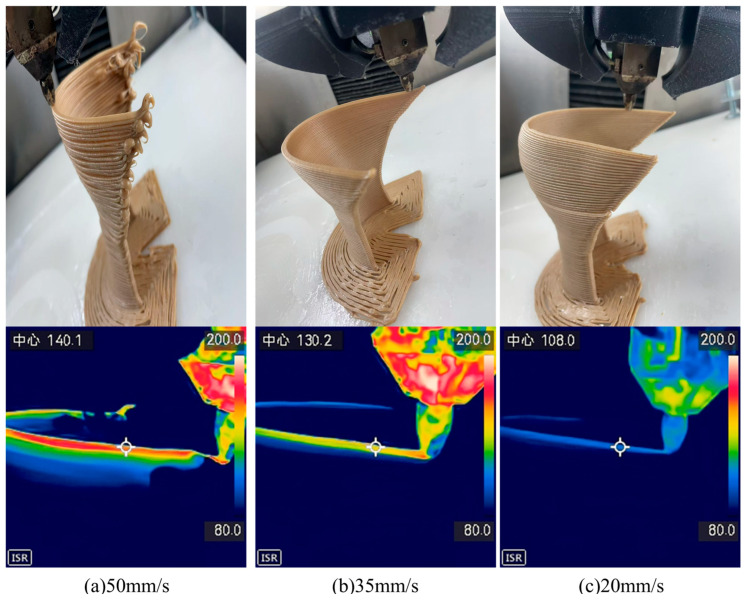
Orthopedic braces images at different printing speeds by using 10 wt.% BF/PLA. Here, Text in the upper left corner refers to the geometric center of the circle indicated by the symbol, and the following number denotes the temperature at that location.

Further analysis of temperature effects revealed that the lower melt temperature of the 5 wt.% fiber composite led to inadequate interlayer adhesion, causing cracking. The 10 wt.% fiber composite, with a higher melt temperature, achieved better interlayer bonding and significantly improved print quality. Based on these findings, the influence of printing speed on fabrication quality was systematically evaluated. Figure 9 and Figure 10 illustrate the printing process and corresponding melt-center temperature profiles for the two composites at different printing speeds (50 mm/s, 35 mm/s, and 20 mm/s).

The experiments showed that at a printing speed of 50 mm/s, the temperature in the central melt region and the residual heat in previously printed layers were significantly higher than under slower conditions, leading to structural collapse. As the speed decreased to 35 mm/s and 20 mm/s, the composite with 5 wt.% bamboo fiber-due to its lower melt temperature-exhibited earlier onset of interlayer cracking, with cracking severity increasing at slower speeds. In contrast, the composite with 10 wt.% bamboo fiber showed cracking only at the slowest speed of 20 mm/s.

This behavior is closely related to the diffusion dynamics of polymer chains. Excessively high printing speeds shorten the contact time between layers, preventing sufficient interdiffusion and bonding of polymer chains before the next layer is deposited, which accumulates thermal stress and results in collapse. Conversely, overly slow printing allows the melt temperature to drop too rapidly, reducing flowability and creating uneven temperature distributions that hinder interlayer heat transfer. This leads to localized residual stress and weakened interlayer adhesion.

**Figure 11:**
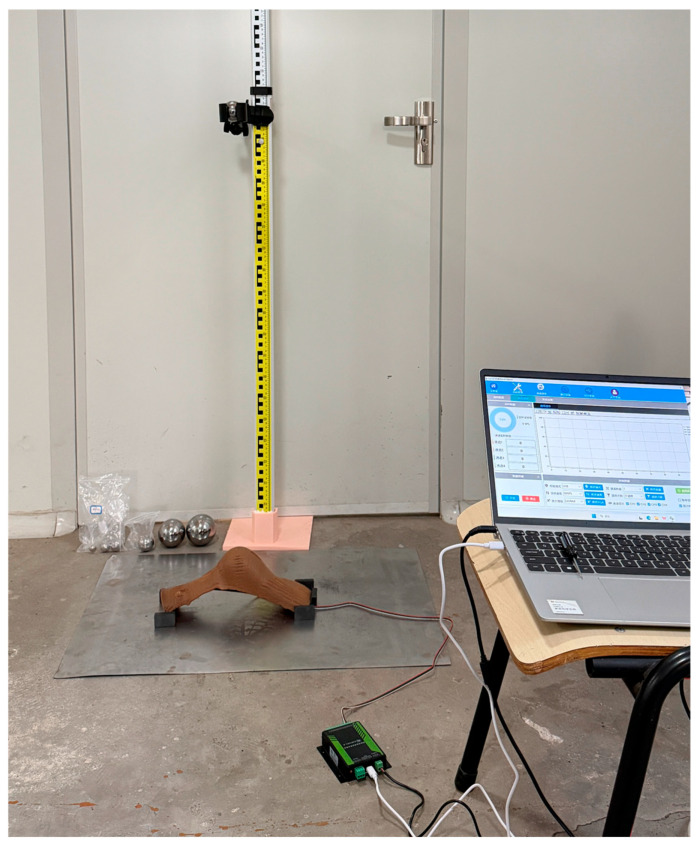
The Drop Ball Impact Platform.

To scientifically evaluate the impact resistance of the 3D-printed brace, a custom test platform based on the drop-weight impact principle was constructed in this study (physical setup shown in Figure 11). The platform mainly consists of a vertically adjustable stand with precise height control and an impact head holder equipped with a release mechanism, ensuring consistent impact location and experimental repeatability. During testing, the foot brace was fixed on the base plate with its outer surface facing upward, simulating impact loading on the brace’s exterior under real-world conditions.

The impact energy was determined based on a mechanical estimation of a typical impact that the foot may sustain during human walking. We considered a scenario in which the foot accidentally strikes a fixed obstacle while walking at a normal speed (approximately 1.4 m/s). The equivalent mass of the lower leg and foot directly involved in the impact was taken as about 4.5 kg, with the velocity assumed to drop to zero immediately after impact. According to the kinetic energy formula, the energy E involved in this impact is given by:

(2) $$E=\frac{1}{2}mv^{2}\;=\;\frac{1}{2}\;\times \;4.5\;\mathrm{kg}\;\times \;{\left(1.4\;m/s\right)}^{2}\;=\;4.41\;J$$

To standardize the test setup and ensure reproducibility, this energy was applied using a 500 g steel impactor in free-fall. Based on the gravitational potential energy formula *E* = *mgh*, the corresponding drop height *h* was calculated as:

(3) $$h\;=\frac{E}{\;mg}\;=\;\frac{4.41\;J}{0.5\;\mathrm{kg}\;\times \;9.8\;m/s^{2}}\;=\;0.9\;m$$

Therefore, to ensure that the developed orthopedic braces meet a fundamental safety threshold, the drop-weight impact test in this study adopted the impact energy generated by a 500 g steel ball freely falling from a height of 0.9 m as the minimum testing criterion. This approach provides an intuitive and effective means to evaluate the ability of brace specimens, printed under different process parameters, to withstand simulated real-world impact energy, thereby supplying product-safety-oriented experimental evidence for process optimization.

**Figure 12:**
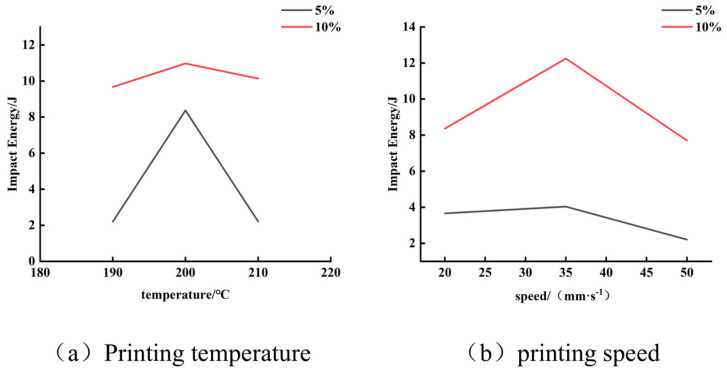
The maximum impact energy variation.

Figure 12 illustrates the variation in the maximum impact energy that the composites containing 5 wt.% and 10 wt.% bamboo fiber, respectively, could withstand under different printing speeds and nozzle temperatures.

As shown in Figure 12, comparing the impact resistance of composites with the two bamboo fiber contents under different printing parameters reveals that—except for the 5 wt.% bamboo fiber composite printed at 200 °C, which withstood a maximum impact energy of 8.374 J (safety factor 1.89) and exceeded the minimum requirement of 4.41 J-all other conditions failed to meet the impact-performance criterion. In contrast, the composite containing 10 wt.% bamboo fiber satisfied the impact requirement under all tested conditions. At a printing temperature of 200 °C, its impact energy reached 10.976 J (safety factor 2.49), and at a printing speed of 35 mm/s, it further increased to 12.25 J, corresponding to a safety factor as high as 2.78, demonstrating excellent impact resistance.

These results indicate that increasing the bamboo fiber content significantly enhances the energy absorption capacity of the composite under impact loading. This improvement is primarily attributed to the more uniform reinforcement network formed by an appropriate fiber content (10 wt.%), which effectively dissipates impact energy through multiple mechanisms such as fiber pull-out, crack deflection, and interfacial debonding. Furthermore, printing speed had a more pronounced influence on impact performance than printing temperature. Moderately reducing the printing speed (e.g., to 35 mm/s) allowed for more complete melt deposition and stronger interlayer bonding, thereby improving the overall integrity and impact resistance of the brace. In comparison, the effect of printing temperature on the final performance tended to stabilize.

Based on the above analysis, the optimal process parameters were determined as follows: using the BF/PLA composite containing 10 wt.% modified bamboo fiber, printed at a speed of 35 mm/s and a nozzle temperature of 200 °C. This combination ensures favorable melt flow and interlayer fusion during deposition, yielding a fully structured foot brace with outstanding impact resistance, thereby providing a critical safety guarantee for its practical application.

## 4. Conclusions

This study developed a high-toughness BF/PLA composite suitable for 3D printing, investigated its application in orthopedic braces, and presents the following conclusions:

At a PLA/PBAT mass ratio of 85/15, the unmodified bamboo fiber-reinforced PLA composite exhibited optimal overall mechanical performance, achieving an impact strength of 7.7 kJ/m^2^. This represents a 275% increase over pure PLA, confirming the significant toughening effect of this matrix composition.

Treating bamboo fibers (BF) with a silane coupling agent further enhanced the impact strength of the composite. A maximum impact strength of 11.3 kJ/m^2^ was achieved with a composite containing 10 wt.% BF treated at a silane-to-fiber mass ratio of 2/98. Scanning electron microscopy revealed that this concentration of KH570 effectively improved the compatibility between PLA and PBAT, reduced fiber agglomeration, and promoted superior interfacial bonding between the fibers and the matrix.

Regarding 3D-printability, the composite containing 10 wt.% bamboo fiber modified with 2 wt.% KH570 demonstrated the best printing adaptability, successfully accommodating a wide printing-temperature window (190, 200, and 210 °C). Further investigation identified printing speed as a more critical parameter than temperature for determining final part quality. An optimal printing speed of approximately 35 mm/s yielded structurally sound orthopedic braces with outstanding impact resistance, capable of absorbing up to 12.25 J of energy and achieving a safety factor of 2.78.

## Figures and Tables

**Table 1:** Composite material ratio.

Samples	PLA/wt.%	PBAT/wt.%	BF/wt.%	KH570/wt.%
PLA	100	0	0	0
P10-BF10	90	10	10	0
P15-BF10	85	15	10	0
P20-BF10	80	20	10	0
P25-BF10	75	25	10	0
P30-BF10	70	30	10	0
K1-BF10	85	15	10	1
K2-BF10	85	15	10	2
K3-BF10	85	15	10	3
K2-BF5	85	15	5	2
K2-BF15	85	15	15	2

**Table 2:** DSC data.

Samples	*T_cc_*/°C	Δ*H_cc_*/J·g^−1^	*T_m_*/°C	Δ*H_m_*/J·g^−1^	*X_c_*/%
PLA	112.3	19.84	174.1	25.26	5.54
P10-BF10	101.4	18.20	173.0	29.76	11.82
P15-BF10	104.9	20.16	172.9	30.14	10.21
P20-BF10	101.3	19.01	172.7	27.94	9.14
P25-BF10	105.1	14.20	172.9	21.84	7.82
P30-BF10	105.7	15.78	172.3	24.48	8.90
K1-BF10	107.9	17.03	173.4	23.71	6.86
K2-BF10	106.2	18.97	172.7	24.76	5.92
K3-BF10	105.8	17.71	172.7	24.32	6.76
K2-BF5	107.6	12.05	175.4	19.48	7.60
K2-BF15	107.7	18.2	173.8	28.12	10.15

*T_m_* = melting temperature; *T_cc_* = cold crystallization temperature.

## Data Availability

The original contributions presented in this study are included in the article. Further inquiries can be directed to the corresponding authors.
